# EGFR-Dependent Extracellular Matrix Protein Interactions Might Light a Candle in Cell Behavior of Non-Small Cell Lung Cancer

**DOI:** 10.3389/fonc.2021.766659

**Published:** 2021-12-15

**Authors:** Sarah Sayed Hassanein, Ahmed Lotfy Abdel-Mawgood, Sherif Abdelaziz Ibrahim

**Affiliations:** ^1^ Biotechnology Program, Basic and Applied Sciences (BAS) Institute, Egypt-Japan University of Science and Technology (E-JUST), Alexandria, Egypt; ^2^ Zoology Department, Faculty of Science, Cairo University, Giza, Egypt

**Keywords:** epidermal growth factor receptor (EGFR), extracellular matrix (ECM), non-small cell lung cancer (NSCLC), integrin receptors, proteoglycans, glycoproteins, matrix metalloproteinases (MMPs), tyrosine kinase inhibitors (TKIs).

## Abstract

Lung cancer remains the leading cause of cancer-related death and is associated with a poor prognosis. Lung cancer is divided into 2 main types: the major in incidence is non-small cell lung cancer (NSCLC) and the minor is small cell lung cancer (SCLC). Although NSCLC progression depends on driver mutations, it is also affected by the extracellular matrix (ECM) interactions that activate their corresponding signaling molecules in concert with integrins and matrix metalloproteinases (MMPs). These signaling molecules include cytoplasmic kinases, small GTPases, adapter proteins, and receptor tyrosine kinases (RTKs), particularly the epidermal growth factor receptor (EGFR). In NSCLC, the interplay between ECM and EGFR regulates ECM stiffness, angiogenesis, survival, adhesion, migration, and metastasis. Furthermore, some tumor-promoting ECM components (e.g., glycoproteins and proteoglycans) enhance activation of EGFR and loss of PTEN. On the other hand, other tumor-suppressing glycoproteins and -proteoglycans can inhibit EGFR activation, suppressing cell invasion and migration. Therefore, deciphering the molecular mechanisms underlying EGFR and ECM interactions might provide a better understanding of disease pathobiology and aid in developing therapeutic strategies. This review critically discusses the crosstalk between EGFR and ECM affecting cell behavior of NSCLC, as well as the involvement of ECM components in developing resistance to EGFR inhibition.

## 1 Introduction

Globally, lung cancer is the foremost cause of cancer-related death, accounting for 2.09 million cases and 1.76 million deaths in 2018, according to GLOBOCAN (1). Two types of lung cancer are known: non-small cell lung cancer (NSCLC) and small cell lung cancer (SCLC), with an incidence rate of 85% and 14%, respectively. According to histological characteristics, NSCLC is divided into lung adenocarcinoma (ADC), squamous cell carcinoma (SqCC), and large cell carcinoma (LCC) (2). Most likely, lung cancer is diagnosed at locally advanced or metastatic stages in 70% of patients, leading to a low 5-year survival rate (15%) (3). Lung cancer metastasis is the primary cause of death in most patients, including metastasis to the brain (20–40%), bones (30–40%); however, the mechanism has yet remained unclear (4, 5). The latest advances in technology have helped determine genetic, epigenetic, and proteomic alterations in different cancers (6). The epidermal growth factor receptor (EGFR) signaling pathway plays a crucial role in NSCLC progression (7, 8).

The EGFR is a transmembrane glycoprotein receptor that belongs to the ErbB family of receptor tyrosine kinases (RTKs). There are four types of EGF receptors (HER1/EGFR/ErbB1, HER2/ErbB2, HER3/ErbB3, and HER4/ErbB4) that comprise a cysteine-rich extracellular ligand-binding domain (LBD), an α-helix transmembrane domain (single-pass), a C-terminal domain, and except HER3, a cytoplasmic tyrosine kinase (TK) domain (8). The EGFR signaling pathway is multifaceted, with more than 13 extracellular ligands. Upon ligand-receptor binding, the dimerization of the receptor either with the same (homodimerization) or another receptor (heterodimerization) of the EGFR family takes place (9, 10). Upon EGFR dimerization, it activates one or more downstream cascades, including the phosphatidylinositol-3-kinase/protein kinase B (PI3K/AKT), mitogen-activated protein kinase (MAPK), extracellular signal-regulated kinase (MEK/ERK), mammalian target of rapamycin (mTOR), and signal transducer and activator of transcription (STAT) pathways through autophosphorylation of the receptor as well as the cytoplasmic protein binding (11, 12). EGFR is normally downregulated after receptor activation by an endocytic pathway, resulting in receptor degradation or recycling. The uncontrolled EGFR pathway induces aberrant signaling linked with many airway illnesses, including extreme airway proliferation, hypersecretion, mucus overproduction, and advanced distal lung fibrosis and cancer (13, 14). Lung SqCC and ADC patients can harbor abnormal EGFR pathway activation and conserved *ErbB1* gene mutations (15) that are approximately 90% in exons 18–21 of its kinase domain, besides an additional 5% denoted to an in-frame deletion in exons 2–7 (13). Tumor extracellular matrix (ECM) composition can play a role in EGFR-dependent lung cancers.

**Figure 1:**
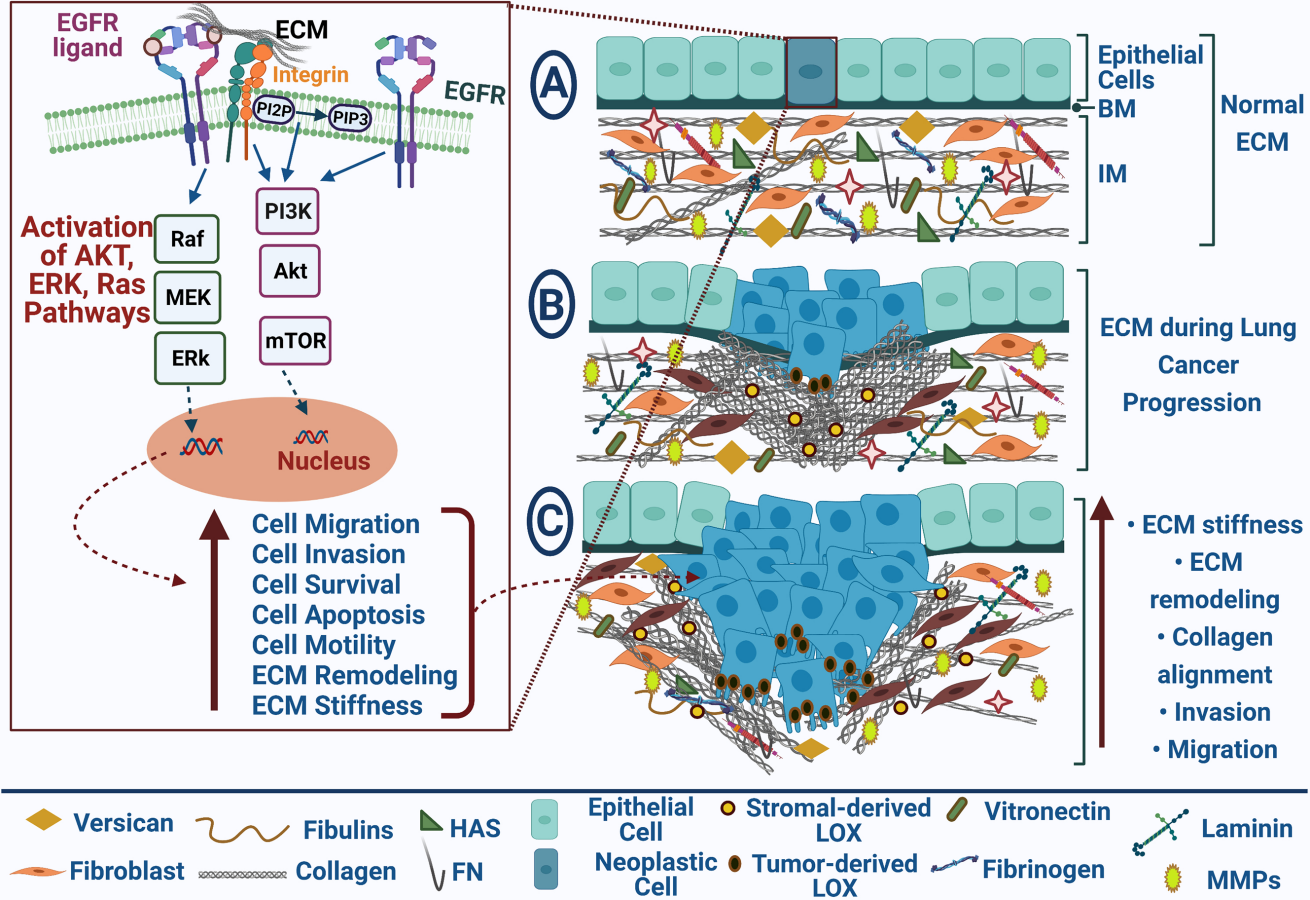
EGFR-mediated ECM remodeling during lung cancer progression. EGFR and ECM receptors, integrins, results in Akt, Erk, and Ras pathways’ activation that participate in increasing cell migration, invasion, survival, and motility and repressing cell apoptosis; **(A)** Normal ECM in healthy tissue; **(B)** Neoplastic cells with uncontrolled cell growth promote ECM remodeling during lung cancer progression; **(C)** Tumor migration and invasion are mediated by collagen alignment and ECM stiffness. Blue arrows point to stimulation, upright-directed red arrows point to increase effect, and dashed red arrows point to cellular effect.

ECM is a significant part of all tissues’ microenvironment. It offers physical support for the neighboring cells, binds growth factors, and controls cell behavior under physiological and pathological conditions (16). ECM is composed of a non-cellular network of proteins, proteoglycans, glycoproteins, and polysaccharides that constitute the interstitial matrix (IM) and the basement membrane (BM) (17). The latter is a well-structured membrane, underlining epithelial and endothelial cells under healthy conditions to separate them from the IM, which constitutes the main stroma and plays a significant role in cell adhesion, cell migration, tissue development, angiogenesis, and repair (18). It is well-known that carcinogenesis is multistep genetic and epigenetic variations, resulting in oncogenes overexpression and downregulation of tumor suppressor genes (19). These aberrations induce cancer cells to stimulate adjacent stromal cells and augment the release of ECM proteins, growth factors, cytokines, angiogenic factors, and proteolytic enzymes into tumor stroma to form a tumor-supportive microenvironment (**Figure 1**) (20, 21). The development of resistance to EGFR tyrosine kinase inhibitors (TKIs) is still a critical problem in lung cancer, and the underlying mechanisms remain fully unexplored (22). Although TKI-induced or –selected genetic alterations are known to cause chemoresistance, other poorly understood mechanisms in tumor cells can drive this resistance. In the absence of genetic alterations, ECM components are players in TKI resistance (23). **I**n the following sections, we highlight the different types of ECM proteins and their roles in mediating EGFR signaling to pinpoint their significance in NSCLC as biomarkers for diagnosis and prognosis and their potential as druggable targets.

## 2 Ecm-Key Structural And Signaling Components Modulate Egfr Activation And Affect Cell Behavior Of NSCLC

### 2.1 Glycoproteins

#### 2.1.1 Fibulins (FBLNs)

Emerging data have indicated that the fibulin (FBLN) family comprising seven members (fibulin-1–7) of widely expressed ECM proteins is associated with lung cancer invasion and metastasis. FBLNs are ECM glycoproteins consisting of EGF-like domain repeats crucial for normal organogenesis and embryonic development (24). They are vital for these biological processes as they regulate cell-to-matrix communication and ECM structure stabilization through intermolecular bridges that bind to several supramolecular structures (25, 26). Besides their structural role, FBLNs are linked to many cellular signaling events and complex biologic processes, including cellular proliferation, adhesion, and migration (25, 27).

**Figure 2:**
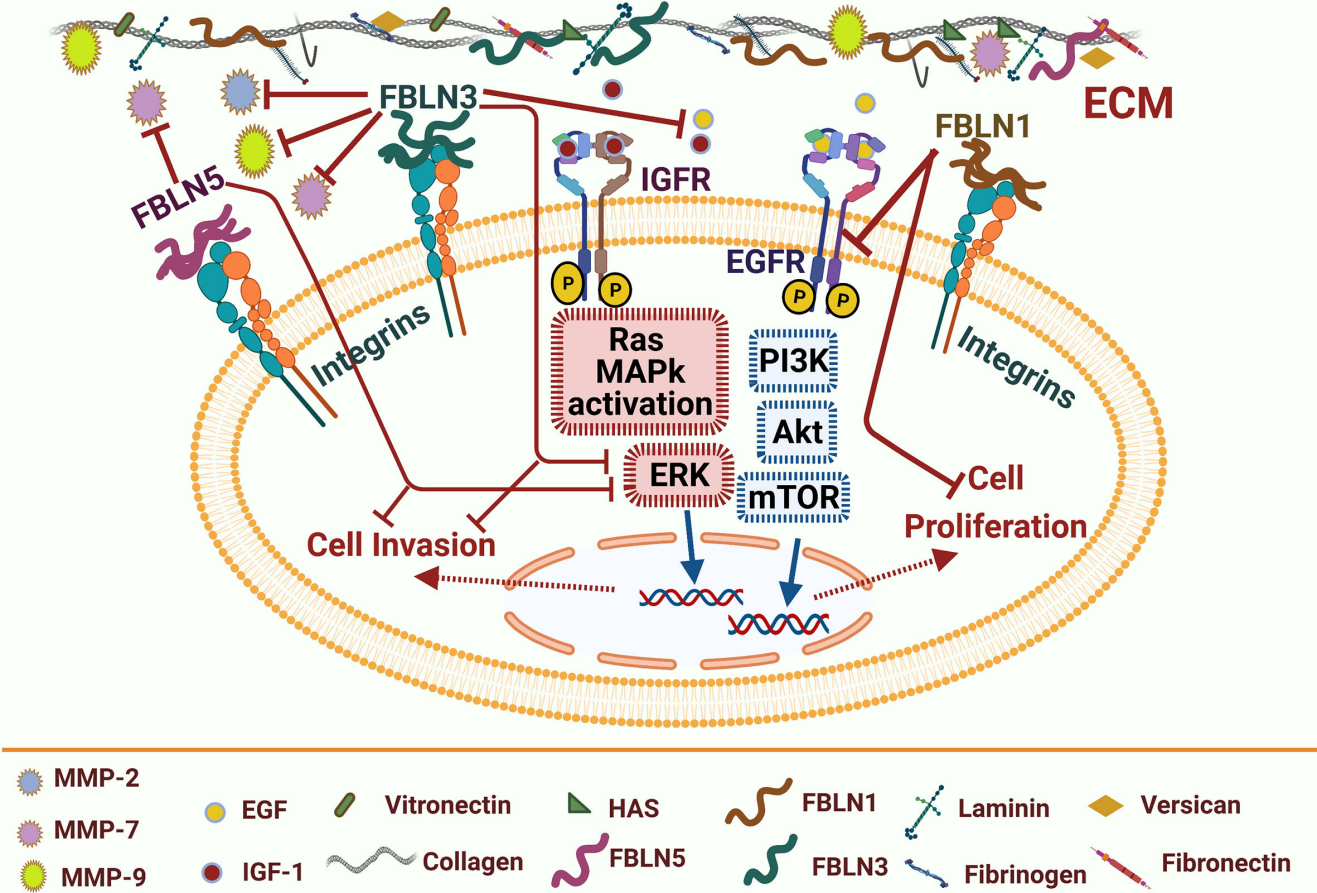
Fibulins-mediated EGFR signaling pathways and matrix metalloproteinases in lung cancer. Fibulin (FBLN) family includes many types such as FBLN1,3&5 serve as tumor-suppressor proteoglycans. FBLN1 can inhibit EGFR activation and thus suppress cell proliferation. FBLN3 can compete with EGF and IGF-1 binding to their receptors; it also can inhibit transcription of oncogenic matrix metalloproteinases (MMP2& MMP9). FBLN3/5 can inhibit MMP7 and Erk pathway activation and thus inhibit cell invasion. Blue arrows for stimulation; dashed red arrows for cellular effect, and red “T” sign for inhibition.

Fibulin-1 (FBLN1) expression levels are substantially downregulated in NSCLC (28). The role of FBLNs in regulating the EGFR function is shown in **Figure 2**. Harikrishnan *et al.* used siRNA to knock down *FBLN1C* and *FBLN1D* expression in NSCLC Calu-1 cells to examine if FBLN1 isoforms could play a role in controlling EGFR signaling and function (28). Without affecting overall EGFR expression levels, *FBLN1C* and *FBLN1D* expression loss significantly increases basal (with serum) and EGF-mediated EGFR activation. Conversely, overexpression of FBLN1D and FBLN1C inhibits EGFR activation, indicating a regulatory crosstalk between the two proteins.

FBLN3’s functions and signaling mechanisms in lung cancer stem cells (CSCs) were investigated (29). Moreover, FBLN3 was downregulated in the lung (30) and nasopharyngeal carcinomas (31). Forced expression of FBLN3 reduces the expression of epithelial-mesenchymal transition (EMT) activators, including N-cadherin and Snail, which inhibit ADC cell invasion and migration. FBLN3 inhibits the stemness activities of ADC cells, as shown by a decline in spheroid formation and the levels of stemness markers, including SRY-like HMG box (Sox2) and β-catenin. FBLN3 effects are mediated by the glycogen synthase kinase-3β (GSK3β)/β-catenin pathway and the upstream regulators of GSK3β such as (PI3K)/AKT and insulin-like growth factor receptor (IGF1R). Furthermore, *IGF1R* was discovered to be a direct target of FBLN*3*, which inhibits the action of IGF. Further, FBLN3 inhibits lung CSC and EMT by modulating the IGF1R/PI3K/AKT/GSK3 pathway, and that FBLN3 may be used as a CSC-centered therapeutic alternative (29). FBLN3 could attenuate the invasion of NSCLC A549 cells by inhibiting the transcription of matrix metalloproteinase- *(MMP)-7* and *MMP-2* (32). Again, Chen et al. revealed the function of FBLN3 and FBLN5 as suppressors of lung cancer invasion and metastasis through the inhibition of Wnt/β-catenin and ERK signaling pathways (33) that, in turn, downregulate MMP-2 and MMP-7 expression (32) and inhibit lung cancer cell survival, proliferation, and metastasis (34, 35). Moreover, FBLN3 overexpression notably decreased the activities of MMP-2 and MMP-9 and repressed the invasion of NSCLC A549 cells; thus, it could be used as a therapeutic strategy for NSCLC (36).

FBLN5 (DANCE), a vascular integrin receptor ligand, is a distinct member of fibulins harboring the RGD (Arg-Gly-Asp) motif associated with endothelial cell adhesion (37). FBLN5 can also depend on RGD to attenuate angiogenesis (38). It interacts directly with elastic fibers *in vitro*, and its amino-terminal domain serves as a ligand for cell surface integrins αvβ3, α9β1, and αvβ5 (39–41). FBLN5 expression is induced under pathological conditions, including pulmonary hypertension and lung injury (42), and is controlled by transforming growth factor-β (TGF-β) (43). FBLN5 was discovered to be a suppressor of lung cancer invasion and metastasis *via* inhibiting MMP-7. Indeed, FBLN5 knockdown induces cell invasion and MMP-7 expression. In lung tumors, the expression levels of FBLN5 and MMP-7 are inversely associated. FBLN5 suppresses MMP-7 expression through the ERK pathway, which is mediated by an integrin-binding RGD motif. FBLN5 overexpression in H460 lung cancer cells also prevents metastasis in mice. These findings indicate that epigenetically silenced *FBLN5* promotes lung cancer invasion and metastasis by inducing MMP-7 expression through the ERK pathway (44).

#### 2.1.2 Mucins

Mucins (MUCs) are high M.wt glycoproteins synthesized by many epithelial tissues (45). They are categorized into two major groups: secretory mucins and membrane-bound mucins. There are 11 membrane-bound mucins (MUC1, MUC3A, MUC3B, MUC4, MUC12, MUC13, MUC15, MUC16, MUC17, MUC20, and MUC21) and seven secreted mucins (MUC2, MUC5AC, MUC5B, MUC6, MUC7, MUC8, and MUC19) (46). MUCs are involved in the normal development of the lungs and are expressed during the embryonic stages of lung development. The cytoplasmic domain of MUC1-C contains i) a YEKV motif: a substrate for EGFR phosphorylation and a SRC SH2 binding site (47), ii) a YHPM motif: a binding site for PI3K and the AKT pathway activation (48, 49), and iii) a YTNP motif: Upon tyrosine phosphorylation, it interacts with Grb2, which binds MUC1-C to son of sevenless (SOS) and thereby activating the RAS→MEK→ERK pathway (49).

MUC1 is an oncogenic glycoprotein that binds to EGFR, serving as a substrate, and that MUC1 expression can enhance EGFR-dependent signaling. MUC1 expression can prevent degradation of EGFR in breast epithelial cells using overexpression constructs and RNAi-mediated knockdown of MUC1, increasing total cellular pools of EGFR (50). The MAPS (MUC1-associated proliferation signature) includes a cytoplasmic domain of MUC1 (MUC1-CD)-dependent genes, including cyclin B1 (*CCNB1*), cyclin-dependent kinase inhibitor 3 (*CDKN3*), cell division cycle protein (*CDC2*, *CDC20*), mitotic arrest deficient 2-like protein 1 (*MAD2L1*), protein regulator of cytokinesis 1 (*PRC1*), and ribonucleoside-diphosphate reductase subunit M2 (*RRM2*), which are involved in cell cycle and proliferation regulation and have been linked to poor outcomes in patients with lung adenocarcinoma (51). MUC1 is expressed as MUC1-N and MUC1-C, a non-covalent heterodimer of N-terminal and C-terminal subunits, respectively (46). MUC1 overexpression, in association with MUC1-C, contributes to activation of the nuclear factor Kappa-activated B cells (NF-κB) (52), Wnt/β-catenin/TCF4 (transcription factor 4) (53), and STAT1/3 pathways in NSCLC (54). In NSCLC, the heterodimeric protein MUC1 is abnormally overexpressed, resulting in gene signatures linked to poor patient survival (48). The cytoplasmic domain of MUC1-C is associated with PI3K p85 in NSCLC cells.

Blocking the interaction of MUC1-C with PI3K p85 *via* cell-penetrating peptides suppresses Akt phosphorylation and its downstream effector mTOR. Treatment of NSCLC cells with GO-203, a MUC1-C peptide inhibitor, results in downregulation of PI3K-Akt signaling, growth inhibition, an increase in reactive oxygen species (ROS), and necrosis induction *via* a ROS-dependent mechanism. Furthermore, in H1975 (EGFR L858R/T790M) mutant cells and A549 (K-Ras G12S) xenografts developed in nude mice after treatment with GO-203, tumor regressions were observed. These data suggest that MUC1-C is needed for PI3K-Akt pathway activation and survival in NSCLC cells (48). Galectin-3 is a β-galactoside binding protein that has also been linked to human cancer development. Glycosylation of the C-terminal subunit of Asn-36 is necessary for galectin-3 upregulation. Two Sentences have been transferred to section no. 8. Galectin-3 binds to MUC1-C at the glycosylated Asn-36 site. Galectin-3 acts as a bridge between EGFR and MUC1, besides galectin-3 is needed for EGF-mediated interactions between MUC1 and EGFR that support the importance of the MUC1-C-galectin-3 interaction (55).

In *EGFR* mutant NSCLC, MUC5B-positive patients had significantly longer overall survival and relapse-free survival than MUC5B-negative patients. MUC5B appears to be a novel prognostic biomarker in NSCLC patients with EGFR mutations (56). Lung ADC subtypes, including invasive mucinous adenocarcinoma (IMA) and lepidic predominant adenocarcinoma (LPA) are associated with MUC expression. In this regard, MUC1 is expressed in LPA, whereas MUC5B, MUC5AC and MUC6 are expressed in IMA (57). Also, *EGFR* and *KRAS* (Kirsten Rat Sarcoma viral oncogene homolog) mutations and Hnf4α expression may participate in mucin expression profiles in these lung ADC subtypes (57). The overexpression of MUC21 proteins with a particular glycosylation state is implicated in developing *EGFR*-mutated lung ADCs associated with a high frequency of lymphatic vessels invasion and lymph node metastasis (58). Additionally, MUC5AC is linked to poor prognosis and would be a prospective therapeutic target in lung ADC due to its role in enhancing tumor heterogeneity with mucin production (59). Therefore, developing treatment strategies targeting MUCs’ expression and functions to manage NSCLC progression are under investigation (60).

#### 2.1.3 Fibronectin

**Figure 3:**
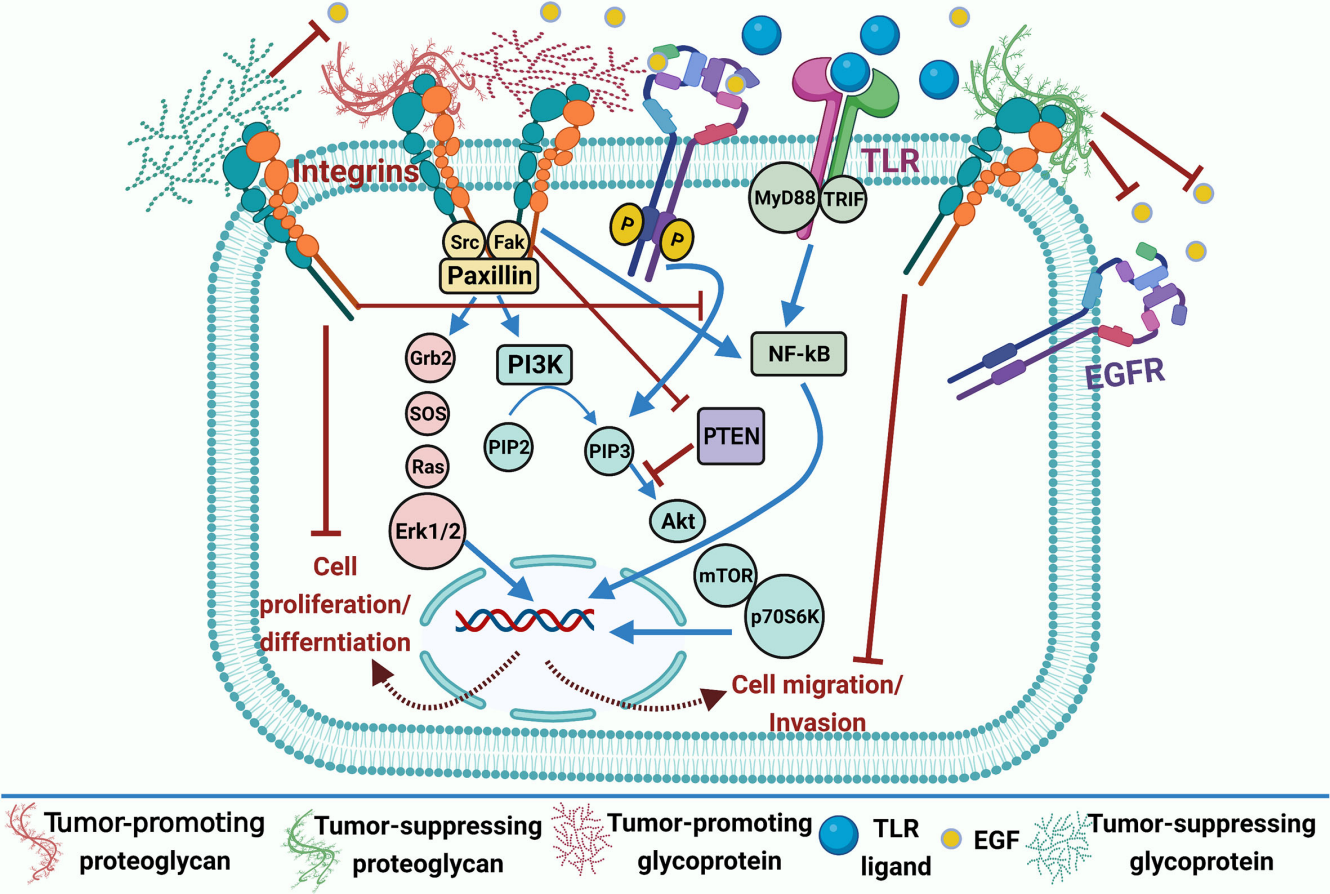
Dual effect of ECM glycoproteins and proteoglycans in lung cancer. Tumor-promoting glycoproteins (e.g., laminin 5 and fibronectin); laminin expression enhances phospho-EGFR or phospho-Akt expression and loss of PTEN; fibronectin activates toll-like receptors (TLRs) to promote NF-κB activation as well as EGFR-dependent Akt/mTOR/p70S6K signaling pathway; and thus, it stimulates cell proliferation and differentiation in lung cancer. Tumor-promoting proteoglycans (e.g., GPC5) prompted cell migration and metastasis. Tumor-suppressing glycoproteins (e.g., fibulins1,3, and 5) compete with EGF and inhibit EGFR activation. Tumor-suppressing proteoglycans (e.g., GPC3 and SDC‐1) can regulate EGF and many intracellular signaling pathways inhibiting cell invasion and migration. Blue arrows for stimulation; dashed red arrows for cellular effect; and red “T” sign for inhibition.

*Fibronectin* (FN) is present in multiple isoforms through alternative splicing, where 20 isoforms in humans have been discovered (61) and are involved in mediating many cellular interactions with the ECM (62). It is primarily synthesized by CAFs and polymerized into ECM fibrils that act as scaffolds for ECM binding molecules such as growth factors and cell surface receptors (63). FN is overexpressed in the stroma of NSCLC and can promote cancer cell adhesion, growth, differentiation, migration, invasion, survival, and resistance to chemotherapy (64). FN-dependent molecular pathways can control the tumor cell response to the stromal matrix and represent potential targets for managing chemo-resistant tumors (65). FNIII-1c, a peptide mimetic, can activate Toll-like receptors (TLRs) to promote NF-κB activation and release inflammatory cytokine in fibroblasts (**Figure 3**) (66, 67). Notably, the PI3K/Akt pathway is the main pathway by which most cytokines and growth factors activate mTOR and its downstream targets. In NSCLC H1838 and H1792 cells, FN induces phosphorylation of eukaryotic initiation factor 4E–binding protein 1 (4E-BP1) and p70S6K1(two downstream targets of mTOR), and Akt phosphorylation (an upstream inducer of mTOR), whereas it inhibits the tumor suppressor protein phosphatase that antagonizes the PI3K/Akt signal (68). Furthermore, FN inhibits liver kinase B1 (LKB1) mRNA and protein expression, as well as the phosphorylation of AMP-activated protein kinase (AMPK), both of which are known to inhibit mTOR. These data indicate that NSCLC cell proliferation induced by FN is mediated by Akt/mTOR/p70S6K pathway activation and LKB1/AMPK signaling inhibition (68).

#### 2.1.4 Laminin

Laminins (Lns) are heterotrimeric extracellular glycoproteins found in all BMs. So far, more than 17 Ln isoforms have been identified with a cross-shaped and specific arrangement of α, β, and γ subunits (69). Ln-332 and Ln5 consist of heterogeneous α3, β3, and γ2 chains and serve as BMs’ essential structural constituent. Ln5 plays a crucial role in cellular migration and tumor invasion (70, 71). NSCLC patients with positive Ln5 expression had a slightly lower survival rate than Ln5-negative expression counterparts. Besides, positive Ln5 expression combined with the loss of PTEN, positive active EGFR expression, or positive active Akt expression has a significantly different overall survival. According to Cox regression analysis, the co-expression of Ln5, PTEN, and p-Akt are the three most independent prognostic markers in NSCLC patients. The findings illustrate the intricate tumorigenesis relationship between key signaling pathway molecules and ECM proteins (71) (**Figure 3**). A Ln receptor, namely integrin α6β4, triggers carcinoma progression through cooperation with various GFRs to facilitate invasion and metastasis (72). Using a lung cancer tissue microarray and immunohistochemistry (IHC), Stewart et al. discovered that SqCC has a higher integrin β4 (*ITGB4*) expression than ADC, and these data were verified in external gene expression data sets. Overexpression of *ITGB4* is also linked to venous invasion and a lower overall patient survival rate. The most highly 50 altered genes related to ITGB4 identified in SqCC were Lns, *CD151*, collagens, *PI3K*, and *EGFR*-associated pathway genes, other recognized signaling partners using cBioPortal. Finally, they show that ITGB4 is overexpressed in NSCLC and is an unfavorable prognostic factor (72). Overall, these data suggest a potential correlation between Lns and EGFR in lung cancer prognosis; however, further studies are still required to profile expression patterns of different Ln types in NSCLC to underscore their clinical relevance.

#### 2.1.5 Fibrinogen

Fibrinogen is a 350 kDa glycoprotein synthesized mainly by the liver epithelium (73). It comprises two similar sets of three polypeptide chains, including Aα, Bβ, and γ, linked by five symmetrical disulfide bridges (74). Many proteins and cytokines such as vascular endothelial growth factor (VEGF) and fibroblast growth factor-2 (FGF-2) bind to fibrinogen affecting its biological behavior (75, 76). Lungs produce fibrinogen by inflammatory stimuli (77). Fibrinogen changed into insoluble fibrin *via* activated thrombin considerably affects blood clotting, inflammatory response, wound healing, fibrinolysis and neoplasia. Increased fibrinogen activity considerably affects cancer cell growth, progression, and metastasis (78).

Accumulating evidence indicates a correlation between fibrinogen and EGFR in lung cancer (79–81). A study by Shang et al. discovered a novel serum protein, fibrinogen alpha chain isoform 2 (FGA2), in lung ADC patients with mutated EGFR using microarray data analysis of 41,472 antibodies coupled with mass spectrometry analysis (79). Further, plasma FGA2 levels were remarkably downregulated in *EGFR*-mutated patients relative to those with the wild-type *EGFR* (81). In the same study, hyperfibrinogenemia was linked to distant metastasis and lymphatic tissue metastasis. A multivariate model based on fibrinogen and smoking history was also used to predict *EGFR* mutation status in NSCLC patients (81). Furthermore, Fibrinogen-like protein 1 (FGL1) is significantly overexpressed in the gefitinib-resistant NSCLC cell line PC9/GR more than in the gefitinib-sensitive NSCLC cell line PC9 with an *EGFR* mutation. However, FGL1 knockdown reduces cell viability, decreases gefitinib IC50, and increases apoptosis in PC9/GR and PC9 cells after gefitinib therapy. FGL1 knockdown in PC9/GR tumor cells increases gefitinib’s inhibitory and apoptosis-inducing effects in a mouse xenograft model. Gefitinib’s possible mechanism for inducing apoptosis in PC9/GR cells includes suppressing FGL1 and activating Poly (ADP-Ribose) Polymerase 1 (PARP1) and caspase 3 pathways. By regulating the PARP1/caspase 3 pathway, FGL1 promotes acquired resistance to gefitinib in the PC9/GR NSCLC cell line. As a result, FGL1 may be a possible therapeutic option for NSCLC patients who have developed resistance to gefitinib (80).

#### 2.1.6 Other ECM Glycoproteins

*Tenascin-C* (TN-C) is a glycoprotein composed of 4 distinct domains interacting with matrix constituents, cell surface proteins, soluble factors, and pathogenic components. TN-C affects pulmonic blood vessel invasions by decreasing apoptosis and promoting cancer cell plasticity, thus, increasing lung metastasis (82). TN-C also binds to more than 25 different molecules, including EGF-L repeats (a low-affinity ligand for the EGFR, MAPK, and phospholipase-C gamma (PLC)-γ signaling). Besides, TN-C binds to FNIII, aggrecan, integrins, and perlecan, along with growth factors such as FGF, platelet-derived growth factor (PDGF), and TGF-β families (83). Again, receptor-type tyrosine-protein phosphatase zeta (PTPRζ1), fibrinogen-like globe (FBG) that can bind to integrins, and TLR4 are TN-C-related molecules (83). These diverse interactions render TN-C a significant driver for many processes such as cell attachment, cell migration, cell spreading, cell survival, focal adhesion, neurite outgrowth, protease, and matrix assembly, and pro-inflammatory cytokine synthesis (83). However, a correlation between TN-C and EGFR has not yet been elucidated in NSCLC.

*Periostin (Postn*, *PN, or osteoblast-specific factor OSF-2)* is a vital ECM protein known for its complex role in tumorigenesis (84). It can directly bind to many ECM proteins, including TN-C, FN, collagen, and Postn itself (85). Also, it acts as a ligand for numerous integrins such as α_v_β_3_, α_v_β_5_, and α_6_β_4_ to participate in cell adhesion, survival, and migration (85, 86). Postn affects tumor progression by regulating cellular survival, angiogenesis, invasion, and metastasis in epithelial tumors (85). Periostin is overexpressed and enhances metastatic growth in colon cancer by inhibiting stress-induced apoptosis in cancer cells and increasing endothelial cell survival to boost angiogenesis. Although there is no direct association between Postn and EGFR in lung cancer, Postn can regulate EGFR interacting partners or its downstream signaling. Periostin increases cellular survival at the molecular level by activating the Akt/PKB signaling pathway through α_v_β_3_ integrins (87). In lung cancer, high Postn expression is positively associated with the EMT markers Snail and Twist and lung cancer stage, according to IHC results. Further, recombinant Postn causes EMT in lung cancer cells through the p38/ERK pathway, and that pretreatment with chemical inhibitors prevents Postn-induced EMT (88). Moreover, the increased Postn expression in the NSCLC A549 cells is one form of cellular response to chemical-mimic hypoxia stress, and this effect can be controlled by hypoxia-inducible growth factors like TGF-α and bFGF, which trigger the RTK/PI3-K pathway leading to upregulation of Postn, and in turn, facilitating the survival of A549 cells in a hypoxic microenvironment *via* the Akt/PKB pathway (89). Collectively, these data indicate that Postn may serve as a therapeutic target in NSCLC.

*Vitronectin* (VTN) is a multifunctional glycoprotein found in blood and ECM. It binds collagen, glycosaminoglycans, the urokinase-receptor, and plasminogen and stabilizes plasminogen activation inhibitor-1 (PAI-1)’s inhibitory conformation. VTN can potentially control the ECM proteolytic degradation through its localization in the ECM and binding to PAI-1. VTN also binds to complement, heparin, and thrombin-antithrombin III complexes, suggesting an immune response role and clot formation control (90). VTN is mostly overexpressed in smaller and well-differentiated tumors (91). EGF promotes carcinoma cell metastasis by phosphorylating p130 CAS in an Src-dependent manner, activating Ras-related protein 1 (Rap1), a small GTPase implicated in integrin activation. Src activity induced by EGFR causes phosphorylation of the CAS substrate, required for Rap1 and αvβ5 activation (92). EGFR activation of Src initiates αvβ5-mediated migration in FG (express stably mutational active Y527F (SrcA) pancreatic carcinoma cells. EGF causes cell metastasis and αvβ5-mediated Rap1 activation. Rac1 and Rap1 activity are increased in FG cells plated on anti-β5, but not anti-β1, integrin antibodies after EGF therapy. Rap1 knockdown on vitronectin but not fibronectin prevents EGF-induced cell migration. In the chick CAM model, knocking down integrin β5 expression prevents EGF-induced pulmonary metastasis but not primary tumor weight (92).

*Nidogen* (NID1 and NID2) are present in the BM and help maintain its stability by connecting COLIV and Ln networks in the ECM (93, 94). The determination of NID2 methylation represents a biomarker for NSCLC diagnosis (95). The lung metastasis of NID1– or 2–deficient mice were studied after being intravenously injected with B16 murine melanoma cells. The authors demonstrated that the depletion of NID2, but not NID1, facilitates melanoma cell lung metastasis. According to histological and ultrastructural examination, the morphology and ultrastructure of BMs, including vessel BMs, are not different in NID1– and 2–deficient lungs. Furthermore, there is no difference in the deposition and distribution of the main BM components between the two mouse strains. These findings indicate that the absence of NID2 can cause subtle changes in endothelial BMs in the lung, allowing tumor cells to move through these BMs more quickly, resulting in a higher risk of metastasis and larger tumors (96). Further, NID2 inhibits liver metastasis in a significant way. NID2 suppresses the EGFR/Akt and integrin/focal adhesion kinase (FAK)/PLC metastasis-related pathways; these data shed light on NID2’s critical tumor metastasis-suppression functions in cancer (97). The roles of NID1 and NID2 in NSCLC have not yet been fully characterized.

### 2.2 Proteoglycans

Proteoglycans, key molecular effectors of cell surface and pericellular microenvironments, perform multiple roles in health and diseases because of their polyhedric structure and ability to interact with ligands and receptors that control neoplastic growth and neovascularization (98, 99). Some proteoglycans, like perlecan, have pro- and anti-angiogenic properties, while others, like syndecans and glypicans, can directly influence cancer growth by modulating key signaling pathways. Several groups of enzymes in the tumor microenvironment further regulate the bioactivity of these proteoglycans: (i) various proteinases, which cleave the protein core of pericellular proteoglycans, (ii) endosulfatases and heparanases which change the structure and bioactivity of various heparan sulfate proteoglycans and their bound growth factors, and (iii) sheddases, which cleave transmembrane or cell-associated syndecans and glypicans. On the other hand, small leucine-rich proteoglycans like lumican and decorin serve as tumor suppressors by physically antagonizing RTK such as EGFR and c-Met (receptor for HGF), evoking antisurvival and proapoptotic pathways (98).

Proteoglycans, including serglycin (100), perlecan (101), versican (102), aggrecans (103), decorin (104), lumican (105), syndecans (106), testicans (107), endocan (108), and glypicans (109) are involved in EGFR signaling pathways in lung cancer (108). For example, endocan is known to be a RTK ligand enhancer in tumorigenesis. Higher endocan levels are observed in lung tumors relative to non-neoplastic tissues, and these levels are associated with a poor prognosis in NSCLC patients with mutant EGFR. Circulating endocan levels are also significantly higher in patients with mutant EGFR than those with wild-type EGFR. Endocan enhances tumor growth driven by mutated EGFR by facilitating EGFR signaling through direct binding and enhancing the EGF-EGFR interaction. Through the Janus kinase (JAK)/STAT3 and ERK/ELK cascades, activated EGFR upregulates endocan expression, creating a positive regulatory loop of endocan-EGFR signaling. These results point to a novel relationship between EGFR and endocan and new strategies to target the endocan-EGFR regulatory axis in NSCLC patients with TKI-resistant (108). Another example is decorin, small leucine-rich proteoglycans (SLRP) that control cell growth and migration in several tumor cell lines (104). Up-regulation of decorin inhibits proliferation, arrests the cell cycle at G1, and reduces invasive activity in the NSCLC A549 cells. Further, upregulating decorin substantially reduces EGFR phosphorylation, cyclin D1, TGF-1 expression and increases *p53* and *P21* expression; whereas, decorin downregulation could reverse the effects (104). In the following section, we discuss the available data for syndecans and glypicans as examples for proteoglycans.

#### 2.2.1 Glypicans

Glypicans (GPCs), a heparan sulfate proteoglycan (HSPG) family, consist of core proteins (60- to 70-kDa), heparan sulfate (HS) chains, and a glycosylphosphatidylinositol linkage (110). There are six known GPCs (GPC1-GPC6) in humans. GPCs participate in cell growth by regulating Wnt (111), development by modifying morphogen gradient formation (112), and other multiple signaling pathways. GPCs are abnormally expressed in multiple types of cancer and are crucial for cancer cell growth and progression. The expression of GPC5 is regulated to control cell growth and differentiation throughout mammalian development (113). Also, genetic variations of *GPC5* may share in the increased risk of never-smokers (114). *GPC5* mRNA and protein levels are overexpressed in A549 and H3255 cells. Using shRNA-mediated knockdown or overexpression of *GPC5*, the migration rates of A549 and H3255 cells transfected with pRNAT-shRNA-*GPC5* are lower than controls employing scratch and transwell assays. Using immunohistochemical staining, the high GPC5 expression level in NSCLC is linked to respiratory symptoms of lung cancer, regional lymph node metastasis, poor differentiation, vascular invasion, and a higher TNM stage. According to the Kaplan-Meier analysis, NSCLC patients with high levels of GPC5 expression have a shorter overall survival time relative to those with low levels of GPC5 expression (115). Conflicting data indicated that GPC5 is downregulated and linked to a poor prognosis in lung ADC tissues. Further, the loss of GPC5 expression is controlled by its hypermethylation, according to de-methylation experiments. GPC5 overexpression inhibits lung cancer cell proliferation, migration, and invasion *in vitro* and slows tumor growth *in vivo*, whereas GPC5 knockdown reverses these effects. Moreover, *via* binding Wnt3a on the cell surface, GPC5 inhibits Wnt/β-catenin signaling, thereby mediating tumor suppressor action (113). Therefore, targeting particular GPCs in the tumor microenvironment that acts as ligands for inducing oncogenic pathways represents an effective cancer therapy strategy (100). Although the functions of GPCs have been assigned in different tumors, including lung (116), colon (117), and breast (118) cancers, esophageal squamous cell (119) carcinoma, and pancreatic ductal adenocarcinoma (120), their interaction with EGFR in NSCLC remains yet to be explored.

#### 2.2.2 Syndecans

The syndecan (SDC) family is a transmembrane protein that possesses HS chains on their extracellular domains (121) and consists of four members (SDC1-SDC4). SDC-1 is frequently misexpressed in cancer and associated with invasion, metastasis, angiogenesis, and dedifferentiation (122–128). SDC-1 acts as a coreceptor for a wide range of growth factors (129), including bFGF and HB-EGF (130). A recent study reported that lung cancer has noticeable SDC-1, yet its expression does not associate with lung cancer patients’ survival rate (121). However, a study by Shah et al. revealed that the expression of either NSCLC subtype classifiers EGFR and SDC-1 determined by tissue microarray is correlated with a 30% reduction in the risk of death. Loss of expression of these histologic classifiers is linked to aggressiveness in lung tumors and a poor prognosis (106). Besides, Zhu and colleagues interestingly reported that NSCLC patients with both a SDC4-ROS1 rearrangement and an activating EGFR mutation might acquire resistance to EGFR-TKIs. Although the coexistence of two driver gene mutations in NSCLC is uncommon, triggering alterations of *EGFR, ROS1, ALK*, and *KRAS* have recently been recorded (131, 132).

### 2.3 Non-proteoglycan Polysaccharides

#### 2.3.1 Hyaluronan (HA)

HA is a plentiful constitute of the pericellular matrix that plays a vital role in regulating tissue homeostasis and cancer progression through its interaction with the cell surface receptor CD44 (133). HA synthesis is controlled by growth factors (e.g., EGF) and cytokines such as IL-1β (133). Three hyaluronan synthases (HAS) isoforms, including HAS1, HAS2, and HAS3, are known. CD44-HA interaction can modulate a variety of intracellular signaling by forming coreceptor complexes with many RTKs (e.g., EGFR) (134) that induce oncogenic pathways involved in cancer cell invasion, migration, and metastasis in the human MCF7 and TamR breast cancer cells (135). HA and CD44 are overexpressed in NHLFs/LCAFs (normal human lung fibroblasts vs. lung cancer-associated fibroblasts), followed by NSCLC cells. In NSCLC cells, exogenous HA somehow rescues the fault in cell proliferation and survival. Further, simultaneous silencing of *HAS2* and *HAS3* or *CD44* suppresses the EGFR/AKT/ERK signaling pathway, cell proliferation, and survival (136, 137). Of note, dual targeting CD44/EGFR by HA-based nanoparticles along with systemic administration of plasmid DNA expressing wild-type (wt-) p53 and microRNA-125b (miR-125b) in a genetically engineered mouse model of lung cancer led to an increase of wild-type *p53* and *miR-125b* gene up to 20-fold associated with elevated caspase-3 and *APAF-1* expression-induced apoptosis; thus it may represent an effective gene therapy for NSCLC (138).

Interestingly, treatment with EGF and IL-1β, either alone or combined with TGF-β in ADC, can stimulate HA production in A549 cell line, where treatment with TGF-β/IL-1β changed cell morphology, induced EMT with altered vimentin and E-cadherin gene expression. Also, *HAS3* overexpression induces HA synthesis, MMP9 expression, EMT phenotype, and MMP2 activities and increases invasion of epithelial ADC cell line H358 (133). Induction of HA in H358 cells and adding exogenous HA in A549 cells significantly improved resistance to EGFR inhibitor Iressa. These results propose that increased HA production can promote EMT and Iressa resistance in NSCLC (133). Thus, regulating HA expression in NSCLC can be a new therapeutic strategy (133). Again, HA is implicated in EMT through EGF or TGF-β1 signaling in lung cancer cell line A549, where TGF-β1 upregulates HAS1, HAS2, and HAS3 expressions and augments CD44 expression interacts with EGFR, leading to the activation of the downstream signaling AKT and ERK pathways (139). On the contrary, pretreatment with HAS inhibitors such as 4-methylumbelliferone (4-MU) can suppress TGF-β1’s impact on the expression of CD44 and EGFR and inhibit the CD44-EGFR interaction. Collectively, these data indicate that HA/CD44 interaction mediated by TGF-β1 transactivates EGFR signaling, resulting in EMT induction in NSCLC cells (139).

### 2.4 Fibrous ECM Proteins

#### 2.4.1 Collagens

*Collagens (COLs)* are the major ECM proteins (up to 30% of the total protein mass) in the human body. They are arranged in a relaxed meshwork and possess elasticity to extreme tensile strength owing to their surrounding proteins like elastin and glycoproteins (140). The individual structure of COLs can also create an intricate network that enables them to interact with each other and the surroundings (141). There are 28 known COL types and divided into specific subgroups according to their supramolecular assemblies, including a) fibrillar-forming COLs: the IM significant components such as COL type I, II, III, V, XI, XXVI, XXVII; b) the network-forming COLs: the main components of basement membrane such as type IV, VIII, X, and XVIII COLs (142). Of note, COLI, COLIII, and COLV are predominantly fibroblasts-derived COLs, while COLIV is mainly expressed by epithelial and endothelial cells (143); c) fibril-associated COLs with interrupted triple helices (FACITs) (e.g., IX, XII, XIV, XVI, XIX, XX, XXI, XXII, XXIV); and d) MACITs (membrane-anchored collagens with interrupted triple helices) such as type XIII, XVII, XXIII, and XXV COLs (144–146). COLIV is upregulated in NSCLC stroma, promoting the *in vitro* impairment of cell apoptosis and multidrug resistance. For example, NSCLC cells expressing COLIV are resistant to cis-platinum (DDP), which is mechanistically attributed to the PI3K pathway (147).

Notably, many studies addressed the significant effect of CAFs in tumorigenesis (148, 149). CAFs are the key players in COL dysregulation and turnover, resulting in desmoplasia (tumor fibrosis), where COLs deposit excessively in the tumor surroundings, crosslink, and linearize, thus increasing tissue stiffness (150). This influences the behavior of the nearby tumor cells and controls cell differentiation, proliferation, migration, gene expression, invasion, metastasis, and survival; thereby, directly affecting the cancer hallmarks (151). Tumor tissue with considerable fibroblast-derived COLs is correlated with poor outcomes (152–154). A study by Li et al. reported that the regulation of autocrine COLI expression for sustaining lung cancer cell growth in 3D cultures with fibroblasts provides a new insight for lung cancer targeted therapy (155). CAFs and other molecules can regulate COLs’ expression in cancer cells, such as transcription factors, mutated genes, receptors, and signaling pathways; these molecules can also affect tumor cell behavior by integrins, RTKs (e.g., EGFR), and discoidin domain receptors (156).

In healthy tissues, the biosynthesis of COLs is highly controlled by a great counterbalance of many enzymes, including MMPs and their inhibitors and lysyl oxidases (LOX) (157, 158). In lung tumors, the stroma comprises a stiffer matrix than normal lung tissues due to more collagen modifications (159) mediated by lysyl hydroxylase-2 or procollagen-lysine, 2-oxoglutarate 5-dioxygenase 2 (PLOD2) enzyme enhancing cell invasion and metastasis (160). Fibrotic collagen is primarily modified by PLOD2. PLOD2 was elevated in NSCLC specimens and was linked to a poor prognosis in NSCLC patients. PLOD2 directly enhances NSCLC metastasis by promoting migration and indirectly by inducing COL reorganization, evident by gain- and loss-of-function experiments and an orthotopic implantation metastasis model. In addition, PLOD2 regulation is achieved by PI3K/AKT-FOXA1 axis. The transcription factor FOXA1 directly binds to the PLOD2 promoter for the transcription of PLOD2. These findings indicated that the NSCLC metastasis mechanism could be regulated by EGFR-PI3K/AKT-FOXA1-PLOD2 pathway and PLOD2 can be a therapeutic target for NSCLC treatment (161).

## 3 Integrins -ECM-Interacting Cell Membrane Receptors

In tumorigenesis, a complex relationship is established between ECM proteins and key signaling pathway molecules (71). Cell–ECM interactions are implicated in the intracellular signals that control gene expression, cell cycle progression, survival, movement, and physical support (162). Notably, these processes are governed by cell surface receptors that bind to ECM proteins called integrins. They are α,β heterodimeric transmembrane proteins implicated in many physiological and pathological processes such as adhesion to ECM, proliferation, survival, differentiation, and migration (163). Some integrins bind to the RGD motif on the ECM proteins, and the specificity of integrin binding to various ECM proteins is determined, partially through other amino acids neighboring the RGD sequence (164). Integrin cytoplasmic tails do not possess a kinase activity but activate specific intracellular non-receptor tyrosine kinases, such as FAK; thus, they recruit the Src kinase (165). Src phosphorylates several FAK-associated proteins, including tensin, paxillin, and the adaptor p130^Cas^ (Crk-Associated Substrate). To some extent, FAK activation results in the recruitment of other SH2-containing proteins, including PLC-γ, PI3K, and the adapter proteins Grb2 and Grb7, mediate ERK activation (165). The FAK/Src complex modulates small GTPase activity, leading to actin cytoskeleton remodeling required for cell adhesion and migration (166).

Upon integrins-ECM binding, numerous signaling molecules are activated, including cytoplasmic kinases, small GTPases, adapter proteins, and growth factor-RTKs (167). The ECM- and EGFR-activated signaling pathways have a high degree of functional interdependence. When EGFR interacts with ECM proteins, autophosphorylation increases in various cell types, including fibroblasts, smooth muscle, and kidney epithelial cells (168, 169)., This type of overlapping signaling is thought to help or improve a variety of ECM and RTK-controlled cell functions, such as proliferation and survival (170). ECM interaction has been discovered to be essential for many EGF-mediated biological responses besides modulating EGFR signaling. EGF, for example, controls integrin-mediated cell migration, an actin-based mechanism that relies entirely on ECM component co-presentation (171).

**Figure 4:**
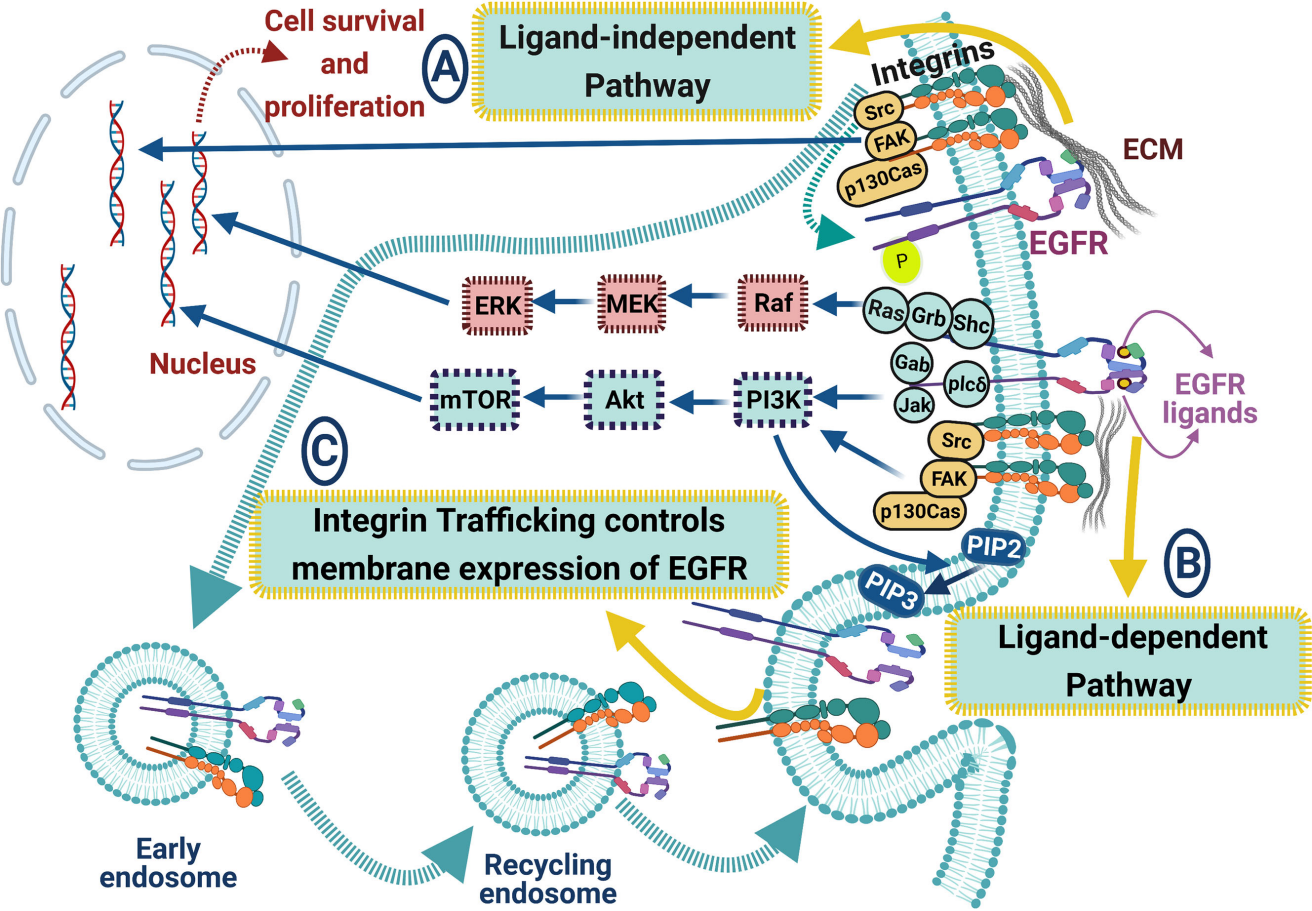
ECM proteins and integrins regulate EGFR signaling pathways in lung cancer. The crosstalk between EGFR and integrins includes many signaling pathways: **(A)** ligand-independent pathway, where integrins can biochemically bind to EGFR leading to its activation. EGFR can interact with integrins via forming a multimeric complex (Src, FAK, and the adaptor protein p130Cas), leading to cell survival and proliferation, **(B)** ligand-dependent pathway, where integrin clustering enhances EGFR signaling cascades upon EGFR ligand binding, resulting in enhancing Akt, ERK, and Ras signaling pathways, and **(C)** integrin trafficking controls the membrane expression of EGFR. Blue arrows for stimulation; dashed red arrows for cellular effect, and red “T” sign for inhibition.

Numerous studies have suggested that integrin-RTK cooperation exists and plays an important role in cancer progression by controlling proliferation, invasion, and survival (172). Various mechanisms could control the crosstalk between integrins and RTKs, regardless of α or β subunit catalytic activity. The integrins’ ability to induce EGFR activation led to the regulation of Erk and Akt activation, which permitted adhesion-dependent induction of p21, cyclin D1 and Rb phosphorylation, and cdk4 activation in epithelial cells in the absence of exogenous growth factors. Epithelial cell adhesion to the ECM fails to efficiently induce p27 degradation, cdk2 activity, or cyclin A and Myc synthesis, and as a result, cells do not progress into the S phase. Treatment of ECM-adherent cells with EGF (to induce EMT), or overexpression of EGFR or Myc, resulted in restoring late-G1 cell cycle events and progression into the S phase. These findings suggest that integrin receptor-mediated partial activation of EGFR is significant in mediating events triggered by epithelial cell attachment to ECM (173). There are three major categories of integrin/RTK interactions (174) (**Figure 4**): (1) Integrins can physically bind to RTKs; (2) integrins clustering upon ECM binding can enhance signaling pathways triggered after ligand-dependent RTK activation, and (3) integrins and RTKs regulate their surface expression in a reciprocal manner (174). EGFR can interact with many integrins in different cancers, such as α6β4 (175), β1 (170), and αvβ3 (176), probably by forming a multimeric complex that also includes Src and the adaptor protein p130^Cas^ (176). This type of interaction is ligand-independent activation of the EGFR, leading to signaling involved in cell survival and proliferation in response to ECM (170) (**Figure 4**).

Emerging data indicate integrins’ importance as essential EGFR signaling regulators in NSCLC (72, 177–179). For example, β1 integrin silencing in human NSCLC A549 cells showed a defective activation of the EGFR signaling cascade, resulting in enhanced sensitivity to Gefitinib and cisplatin, reduced migration, and invasive behavior, and decreased *in vitro* proliferation and *in vivo* tumor growth. This silencing also increases the amount of cell surface EGFR, implying that β1 integrin is required for efficient constitutive EGFR turnover at the cell membrane. Despite having no effect on the EGF internalization rate and recycling in silenced cells, EGFR signaling is recovered only by the Rab-coupling protein (RCP) expression, suggesting that β1 integrin maintains the endocytic machinery required for EGFR signaling (177). Also, Integrin β4 (ITGB4) expression is overexpressed in SCC compared with adenocarcinoma and associated with the presence of venous invasion, low overall patient survival. Using cBioPortal, a network map demonstrates the 50 most highly altered genes neighboring ITGB4 in SCC, which included genes in the EGFR and PI3K pathways and other known signaling partners as well as laminins, collagens, and CD151 (72). Moreover, CD151 drives cancer progression depending on integrin α3β1 through EGFR signaling in NSCLC. In detail, a high CD151 mRNA expression level is detected in NSCLC tissues and cell lines, and its high expression was substantially related to the poor prognosis of NSCLC patients. Also, CD151 knockdown *in vitro* suppressed tumor proliferation, migration, and invasion. Further, overexpression of CD151 enhanced NSCLC growth in a mice model. NSCLC cells overexpressing CD151 exhibit migratory and invasive phenotype via interacting with integrins and regulating the downstream signaling pathways of EGFR/ErbB2 (179). Interestingly, the inhibition of EGFR in NSCLC cell lines reduces tyrosine phosphorylation of neural precursor cell expressed, developmentally down-regulated 9 (NEDD9), an integrin signaling adaptor protein that consists of multiple domains serving as substrate for various tyrosine kinases. Overexpression of constitutively active EGFR, in the absence of integrin stimulation, leads to tyrosine phosphorylation of NEDD9, which plays a pivotal role in the *in vitro* cell migration and invasion of NSCLC cells. Moreover, NEDD9 overexpression promoted lung metastasis of an NSCLC cell line in NOD/Shi-scid, IL-2Rγ(null) mice (NOG) mice (178). Overall, these data show that integrins-dependent EGFR interactions might represent a prognostic marker and potential therapeutic targets in NSCLC.

## 4 Key ECM Remodeling Enzymes

Matrix degradation is a finely regulated process that occurs simultaneously with the formation of new ECM molecules. Tissue integrity is achieved through the actions of matrix-degrading enzymes such as matrix metalloproteinases (MMPs) and their endogenous inhibitors (TIMPs), adamalysin group (ADAMs and ADAMTS), cathepsins, plasminogen activation system components, and glycolytic enzymes such as heparanase (HPSE) and hyaluronidases (HYALs) that cleave heparan sulfate (HS)/heparin chains on hyaluronan (HA) and proteoglycans (PGs) (180, 181). Elastase, dipeptidyl peptidase IV (DPPIV), and tissue kallikrein are ECM serine proteases that play distinct functions in matrix proteolysis and have been linked to cancer progression (182–184). MMPs are the major catabolic matrix endopeptidases linked to a number of normal processes such as wound healing, immunological response, differentiation, tissue homeostasis, and diseases such as osteoarthritis neuroinflammation, atherosclerosis, and cancer (185). The human genome contains 24 MMP members, which are classified into secreted and membrane-bound MMPs. MMPs are categorized as matrilysins, gelatinases, furin-activated collagenases, stromelysins, and other MMPs based on substrate specificity (186, 187). MMPs are mediators of the tumor microenvironment alternations during cancer growth because they enhance EMT, cancer cell signaling, migration, invasion, autophagy, and angiogenesis, which aid tumor progression and metastasis (188). We focus on the functional interplay between MMPs and EGFR in NSCLC in the next sections.

### 4.1 Metalloproteinases (MMPs)

MMPs are a group of 24 proteinases, also known as matrix MMPs, matrixins, and zinc-dependent endopeptidases (189). Transcription of most matrixins is regulated by growth factors, hormones, cytokines, and cellular transformation. MMPs’ proteolytic activities are tightly controlled during their activation from their precursors and inhibition by the endogenous inhibitors TIMPs and a-macroglobulins (190). Aberrant expression of MMPs is associated with many diseases, including lung cancer (191). MMPs perform their proteolytic activity autonomously in the alveolar space for any changes in the cleaved protein properties (192). Cancer cells secrete many MMPs that remodel and degrade the BM in lung cancer tissue, creating a dynamic flow of pro- and antitumor signals (190, 191). The regulation of MMPs expression occurs by triggering inflammatory molecules and hormones and intercellular and matrix interactions (190). MMPs are present in low levels in normal adult tissues, yet the MMPs expression is upregulated during wound healing, tissue repair, or remodeling under pathogenic conditions (192).

When the ECM collagen becomes abundant, large amounts of MMPs are secreted in tumor tissues, and BM remodeling occurs (193, 194), leading to complex chaos of pro-and antitumor signals originating from BM degradation products and enhancing the invasive phenotype of malignant cells (195). In both mouse and human NSCLC, MMP14 is significantly upregulated in intratumoral myeloid compartments and tumor epithelial cells. In an orthotopic (K-Ras^G12D/+^p53^-/-^) mouse model of lung cancer, overexpression of a soluble dominant-negative MMP14 (DN-MMP14) or pharmacological inhibition of MMP14 blocks the invasion of lung cancer cells in collagen I matrix *in vitro* and reduces tumor incidence. MMP14 activity also triggers the proteolytic processing and activation of Heparin-Binding EGF-like growth factor (HB-EGF), which stimulates the EGFR signaling pathway and increases tumor proliferation and growth. These data pinpoint the potential for developing therapeutic strategies that target MMP14 in NSCLC, specifically targeting the MMP14-HB-EGF axis (191). Increased expression of MMP-9 *in vitro* and *in vivo* has been linked to tumor progression. Cox et al. linked the EGFR expression with MMP-9 upregulation in tumor cells *in vitro* in NSCLC patients. MMP-9 expression strongly correlated with EGFR expression and EGFR membranous expression, but not with cytoplasmic EGFR expression. MMP-9 and EGFR co-expression is associated with a poorer prognosis in NSCLC patients. Also, MMP-9 and EGFR are expressed in a large proportion of NSCLC tumors. The presence of these markers together indicates a poor prognosis. These findings suggest that the EGFR signaling pathway, *via* specific up-regulation of MMP-9, can play a key role in NSCLC invasion (196).

## 5 Effect Of ECM Components’ Expression and Interactions on Sensitivity to TKI Therapy 

The biological features of the tumor microenvironment are affected by cancer cells, non-cancerous cells, and ECM (197). The interactions between different cell types within the tumor microenvironment play a key role in developing resistance to the anticancer drugs (198). The most abundant matrix protein in the cancer stroma, COLI, promotes tumor progression by facilitating cancer cell growth, invasion, and metastasis (199, 200). Also, COLI supports anticancer drug resistance through the integrin signaling pathway (201). Besides, COLI induces EGFR-TKI resistance in EGFR-mutated cancer cells (202). Moreover, the results of Wang *et al.* reported that COLI drives EGFR-TKI resistance through integrin-β (23). Knockdown of integrin-β1 significantly suppresses the resistance driven by both COLI and de-cellularized ECM, indicating that COLI and integrin-β1 could mediate the resistance-driving function of ECM and might be useful interventional therapeutic strategies. Further, a collagen synthesis inhibitor, CHP (cis-4-Hydroxy-L-proline), efficiently inhibiting collagen production and synergizing with osimertinib, leads to growth suppression of GFP-labeled H1975 cells co-cultured with parental H1975 cells or fibroblasts (23). Interleukin-6 (IL-6) plays a vital role in developing interstitial fibroblastic proliferation induced by EGFR-TKI. In lung cancer, A549 cell lines treated with EGFR-TKIA reduce cell viability *via* increment of IL-6 mRNA and protein expression. IL-6 treatment increases α-actin and collagen expression, fibrosis markers, in lung fibroblast cells using a co-culture model. These findings indicate that IL-6 plays a role in EGFR-TKI-induced interstitial fibroblastic proliferation. Therefore, inhibiting IL-6 could be helpful to cancer patients receiving EGFR-TKI treatment to reduce the risk of side effects (203). Further, in EGFR mutated lung ADC patients, FG2A level was related to EGFR-TKI response, and FGA2 represented a predictor of targeted therapy for EGFR-mutated lung (79). Furthermore, integrin β1 promotes Src-Akt pathway activation and induces erlotinib resistance (201). COLI is dysregulated in the bone, and other solid tumors influence tumor cell behavior inducing EMT, including the lung (204) and breast (205). The sensitivity of EGFR-TKI in EGFR-mutated cancer cells cultured with COLI was investigated when COLI activated mTOR *via* Akt and ERK1/2-independent pathway in NSCLC, leading to EGFR-TKI resistance. Combining EGFR-TKI and mTOR inhibitors may be a viable option for combating such resistance (206). ECM components are internalized and used as nutrients by cells through several mechanisms. For example, the degradation of ECM proteins into peptides by MMPs and internalization of the degraded peptide fragments by cells. Another mechanism involves endocytosis of ECM macromolecules (207, 208). Rac1 inhibition reduces COLI uptake in mutated lung cancer cells (PC-9) and restores their sensitivity to EGFR-TKI. Rac1 is needed for micropinocytosis and reduction of COLI uptake. Thus, EGFR-TKI resistance can evolve in EGFR-mutated lung cancer cells *via* COLI uptake mediated by micropinocytosis (202).

EMT is characterized by the downregulation of epithelial markers, especially E-cadherin, and upregulation of mesenchymal markers such as vimentin, N-cadherin, and fibronectin (209). EMT is essential in the primary resistance of erlotinib in the EGFR-TKI responsive EGFR-mutant lung cancer cell line (210, 211). The expression of EGFR and EMT-related proteins are noticeably modulated in the peripheral leading edge of NSCLCs associated with poor prognosis (212). In NSCLC, EMT is a key player in controlling sensitivity or resistance to EGFR inhibition. NSCLC lines expressing E-cadherin showed higher sensitivity to EGFR inhibition *in vitro* and xenografted models, whereas NSCLC lines expressing vimentin and/or fibronectin showed resistance to the growth inhibitory effects of EGFR kinase inhibition (210).

FBLN1 isoforms regulate EGFR signaling and function in NSCLC. FBLN1 loss, using siRNA mediated knockdown of FBLN1C and FBLN1D, in NSCLC Calu-1 cells significantly increased EGF mediated EGFR activation, inhibited EGFR activation, promoted EGFR-dependent cell migration that inhibited upon Erlotinib treatment. Notably, FBLN1C and FBLN1D knockdown cells show a substantial increase in EGF-mediated EGFR activation, which promotes cell adhesion reduced by Erlotinib treatment. These data point out that FBLN1C/1D, as an ECM protein, can bind and regulate EGFR function and activation in NSCLC Calu-1 cells, highlighting tumor ECM role in affecting EGFR dependent lung cancers (6). In H1975/EGFR (L858R/T790M) cells, stable silencing of MUC1-C downregulates AKT signaling and inhibits colony formation, growth, and tumorigenicity. Similar results were found during MUC1-C silencing in gefitinib-resistant PC9GR cells that express EGFR (delE746_A750/T790M). Further, inhibition of MUC1-C suppresses the activation of EGFR (T790M), AKT, ERK, and MEK activation, colony formation, and tumorigenicity. Treatment of PC9GR and H1975 cells with GO-203 inhibits MUC1-C homodimerization, results in EGFR, AKT, and MEK/ERK signaling inhibition, as well as loss of survival. The combination of GO-203 and the irreversible EGFR inhibitor afatinib acts in synergism to inhibit the growth of NSCLC cells harboring activating EGFR (T790M) or EGFR (delE746-A750) mutants (213).

The activation of many signaling pathways imperils the clinical efficacy of EGFR-TKIs in EGFR-mutated NSCLC (214–218). The interactions between tumor cells and the extracellular environment are regulated by an integrin-linked kinase (ILK) to promote cell proliferation, migration, and EMT. Src homology 2 domain-containing phosphatase 2 (SHP2) is essential for MAPK pathway and RTK signaling activation. In baseline tumor specimens, highly expressed ILK mRNA is associated with poor prognostic factors for patient-free survival in the univariate and multivariate Cox regression models (214). Integrin β3 was significantly and consistently overexpressed in acquired osimertinib- or gefitinib-resistant lung cancer *in vitro* and *in vivo* and involved in the progression of lung ADC. Antagonizing integrin β3 improved the TKI sensitivity *in vitro* and *in vivo*, inhibiting anoikis resistance, proliferation, and EMT phenotype in lung cancer cells. Integrin β3 overexpression was also linked to the enhanced cancer stemness implicated in resistance development. Mechanistically, integrin β3 is induced by increased levels of TGFβ1 in acquired TKI-resistant lung cancer, which indicates the TGFβ1/integrin β3 axis as a potential target for combination therapy in EGFR-mutant lung cancer to overcome acquired resistance to EGFR TKIs (215). Furthermore, azurin, an anticancer therapeutic protein, controls integrin β1 levels, and its appropriate membrane localization suppressed the intracellular downstream signaling cascades of integrins and the invasiveness of NSCLC A549 cells. Further, azurin combined with erlotinib and gefitinib enhances the sensitivity of NSCLC A549 cells to azurin. The stiffness of A549 lung cancer cells decreased with exposure to azurin and gefitinib using Young’s module (E), suggesting that the changes in the membrane properties are the principal of the broad anticancer activity of azurin, and it may be relevant as an adjuvant to enhance the effects of other clinical anticancer agents (216). The expression levels of EGFR and integrin α2 and β1 subunits were significantly elevated in Ionizing radiation (IR) cells. Importantly, functional blockade of integrin α2β1 or treatment with EGFR-TKI, PD168393, resulted in a round morphology of cells and revoked their invasion in the collagen matrix. Further, higher activation of Erk1/2 and Akt signaling molecules in IR cells. Inhibition of Akt activation by treating with PI3K inhibitor LY294002 decreased IR cell invasion, yet MEK inhibitor U0126 did not inhibit Erk1/2 activation, which indicates integrin α2β1 and EGFR mutually promote higher invasiveness mediated by the PI3K/Akt signaling pathway in IR-survived lung cancer cells and might provide alternative targets along with radiotherapy (217). Recently, EGFR inhibitors’ resistance was delayed by co-delivering EGFR and integrin αvβ3 inhibitors with nanoparticles in NSCLC. The enhanced expression of integrin αvβ3 is observed in tumor tissues of patients resistant to EGFR inhibitors. Further, integrin αvβ3-positive NSCLC cells unveiled significant EGFR inhibitor resistance, leading to activating Galectin-3/KRAS/RalB/TBK1/NF-κB signaling pathway. Interestingly, co-encapsulating erlotinib and cilengitide by MPEG-PLA (Erlo+Cilen/PP) nanoparticles enhanced the drug delivery system, leading to reduced systemic toxicity and superior anti-cancer effects (218).

## 6 Conclusion and Future Perspectives

ECM components, along with integrin and MMPs, regulate many cellular processes relevant to lung cancer progression, including cell proliferation, adhesion, and migration through their direct or indirect interactions with EGFR. ECM proteins associated with poor NSCLC prognosis *via* crosstalk with EGFR, including COLs, MMP-9, MUC1, MUC5AC, Ln 5, and GPC5. Many ECM proteins can be used as therapeutic targets, such as COLs, PLOD2, FBLN3, MUC5AC, FN, FAG2, FG, GPC3, and HA by modulating their interaction with EGFR. ECM proteins can be tumor-suppressing or -promoting depending on their signaling context with EGFR and many signaling molecules. Despite the emerging data revealing the role of ECM components or/and EGFR in NSCLC, many gaps still exist in EGFR-ECM interactions. The correlation between EGFR and many ECM proteins, including COLI, COLIV, FBLN1, FBLN3, FBLN5 MUC1, MUC5, MUC6, and Ln5 was revealed, yet EGFR interactions with other types of COLs, MUC and Ln, FG, TN, Postn, VTN, NID, TSP, and versican in NSCLC still need further investigations. A future better understanding of the interactions between ECM components and EGFR-TKI might provide new insights for developing new therapeutic strategies for NSCLC patients.

## Author Contributions

Conceptualization, SH and SI. Literature searches, SH and SI. Data curation, SI, SH, and AA. Investigation, SH, SI, and AA. Supervision, AA and SI. Figures design, SH. Writing—original draft, SH and SI. Writing—review and editing, SH, SI, and AA. This article is based primarily on the student MSc. thesis, SH. All authors have read and agreed to the published version of the manuscript.

## Conflict of Interest

The authors declare that the research was conducted in the absence of any commercial or financial relationships that could be construed as a potential conflict of interest.

## Publisher’s Note

All claims expressed in this article are solely those of the authors and do not necessarily represent those of their affiliated organizations, or those of the publisher, the editors and the reviewers. Any product that may be evaluated in this article, or claim that may be made by its manufacturer, is not guaranteed or endorsed by the publisher.

## Glossary

ADC: Adenocarcinoma
AKT: Protein Kinase B
ALK: Anaplastic Lymphoma Kinase
AMPK: 5’ Adenosine Monophosphate-activated Protein Kinase
APAF-1: Apoptotic Peptidase Activating Factor 1
BM: Basement Membrane
CAFs: Cancer-Associated Fibroblasts
CCNB1: Cyclin B1
CD151: Cluster of Differentiation 151
CDC: cell division cycle protein
CDKN3: Cyclin-dependent kinase inhibitor 3
COLs: Collagens
CSCs: Cancer Stem Cells
Cdk4: cyclin-dependent kinase 4
ECM: Extra Cellular Matrix
EGF: Epidermal Growth Factor
EGFR: Epidermal Growth Factor Receptor
EMT: Epithelial-Mesenchymal Transition
ERK: Extracellular Signal-regulated Kinase
FACITs: Fibril-Associated COLs with Interrupted Triple helices
FAK: Focal Adhesion Kinase
FBG: Fibrinogen-like Globe
FBLN: Fibulin
FGA2: Fibrinogen Alpha chain isoform 2
FGF: Fibroblast Growth Factor
FN: Fibronectin
FOXA1: Fork-head box protein A1
GFRs: Growth Factor Receptors
GPC: Glypican
Grb2: Growth factor Receptor-Bound protein 2
HA: Hyaluronan
HAS: Hyaluronan Synthases
HB-EGF: Heparin-Binding EGF-like growth factor
Hnf4α: Hepatocyte Nuclear Factor 4α
HSPG: Heparan Sulfate Proteoglycan
IGF1R: Insulin-like Growth Factor 1 Receptor
IL-1β: Interleukin-1β
IL-6: Interleukin 6
IM: Interstitial Matrix
IMA: Invasive Mucinous Adenocarcinoma
ITGB4: Integrin Subunit Beta 4
JAK: Janus kinaes
KRAS: Ki-ras2 Kirsten Rat Sarcoma viral oncogene homolog
LPA: Lepidic Predominant Adenocarcinoma
LCC: Large Cell Carcinoma
LKB1: Liver Kinase B1
lncRNA: Long noncoding RNA
Ln: Laminins
LOX: Lysyl Oxidases
MACITs: Membrane-Anchored Collagens with Interrupted Triple helices
MAD2L1: Mitotic Arrest Deficient 2-Like Protein 1
MEK: MAPK Kinase
MMPs: Matrix Metalloproteinases
mTOR: Mammalian Target of Rapamycin
MUCs: Mucins
NF-κB: Nuclear Factor Kappa-light-chain-enhancer of activated B cells
NID: Nidogen
NSCLC: Non-small Cell Lung Cancer
OSF-2: Osteoblast-Specific Factor-2
PARP1: Poly(ADP-Ribose) Polymerase 1
PDGF: Platelet-Derived Growth Factor
PI3K: Phosphatidylinositol-3-Kinase
PKB: Protein kinase B
PLC-γ: Phospholipase C-gamma
PLOD2: Procollagen-Lysine, 2-Oxoglutarate 5-Dioxygenase 2
Postn: Periostin
PRC1: Protein Regulator of cytokinesis 1
PTEN: Phosphatase and Tensin homolog
PTPRζ1: Receptor-type Tyrosine-Protein phosphatase Zeta
p130^Cas^: p130 Crk-Associated Substrate
p70S6K: 70 kDa ribosomal protein S6 kinase
Rac1: Ras-related C3 botulinum toxin substrate 1
Rap1: Ras-related protein 1
ROBO2: Roundabout homolog 2
ROS1: ROS proto-oncogene 1
RRM2: Ribonucleoside-Diphosphate Reductase Subunit M2
RTKs: Receptor Tyrosine Kinases
SCLC: Small Cell Lung Cancer
SDC: Syndecan
SLRP: Small Leucine-Rich Proteoglycans
Sox2: SRY-like HMG box
SOS: Son of Sevenless
Src: Sarcoma viral oncogene
STAT: Signal Transducer and Activator of Transcription
SqCC: Squamous Cell Carcinoma
TCF4: Transcription factor 4
TGF-β: Transforming Growth Factor-β
TKIs: Tyrosine Kinase Inhibitors
TN-C: Tenascin-C
TLRs: Toll-like receptors
TSP: Thrombospondin
VTN: Vitronectin
